# β-endorphin suppresses ultraviolet B irradiation-induced epidermal barrier damage by regulating inflammation-dependent mTORC1 signaling

**DOI:** 10.1038/s41598-023-49886-5

**Published:** 2023-12-15

**Authors:** Hyung-Su Kim, Hyoung-June Kim, Yong-Deog Hong, Eui Dong Son, Si-Young Cho

**Affiliations:** Amorepacific Research and Innovation Center, Yongin, Gyeonggi-do 17074 Korea

**Keywords:** Cell biology, Cell signalling, Stress signalling

## Abstract

Solar ultraviolet B (UVB) radiation triggers excessive inflammation, disrupting the epidermal barrier, and can eventually cause skin cancer. A previous study reported that under UVB irradiation, epidermal keratinocytes synthesize the proopiomelanocortin-derived peptide β-endorphin, which is known for its analgesic effect. However, little is known about the role of β-endorphin in UVB-exposed skin. Therefore, in this study, we aimed to explore the protective role of β-endorphin against UVB irradiation-induced damage to the skin barrier in normal human keratinocytes (NHKs) and on a human skin equivalent model. Treatment with β-endorphin reduced inflammatory responses in UVB-irradiated NHKs by inactivating the NF-κB signaling pathway. Additionally, we found that β-endorphin treatment reversed UVB-induced abnormal epidermal proliferation and differentiation in NHKs and, thus, repaired the skin barrier in UVB-treated skin equivalents. The observed effects of β-endorphin on UVB-irradiated NHKs were mediated via blockade of the Akt/mTOR signaling pathway. These results reveal that β-endorphin might be useful against UVB-induced skin injury, including the disruption of the skin barrier function.

## Introduction

The epidermis, the outermost layer of the skin, protects the human body from pathogenic infections, water loss, and physical injuries by serving as a physiological barrier. To ensure the maintenance of a functional epidermis as a barrier between the human body and the external environment, epidermal homeostasis between differentiation and proliferation should be maintained. To this end, keratinocyte stem cells divide asymmetrically—some remain in the basal layer to maintain their niche; others depart from the basal layer and differentiate into the spinous, granular, and cornified layers, defined by the expression of keratins (including keratin 1 and keratin 10) and other markers, such as loricrin, filaggrin, or involucrin^1^. When keratinocytes mature, they undergo programmed cell death and are ultimately keratinized and released as corneocytes to the outer surface of the skin^2^. The proper balance between the proliferation and differentiation of keratinocytes should be firmly regulated; disruption of skin homeostasis can occur in various skin diseases, such as psoriasis, atopic dermatitis, and several keratoses^3–5^.

In skin disorders, such as atopic dermatitis and psoriasis, the disruption of epidermal homeostasis arises predominantly from irregular inflammatory reactions. Atopic dermatitis skin is characterized by the overexpression of interleukin (IL)-4 and IL-13, which attenuate the expression levels of various components, including involucrin, filaggrin, and loricrin, which regulate epidermal cellular structures and the barrier function^6,7^. Psoriasis is a quintessential immune-mediated inflammatory disorder, marked by heightened levels of proinflammatory markers and cytokines, including, but not limited to, tumor necrosis factor (TNF)-α, IL-6, IL-17, IL-22, and IL-23^8,9^. These proinflammatory cytokines drive epidermal hyperplasia and abnormal keratinocyte differentiation, leading to marked thickening of the stratum corneum and a disturbed skin barrier^10^.

In addition to inflammation prompted by pathological conditions, a range of environmental factors actively modulate the inflammatory responses that impact the skin barrier. Exposure to solar ultraviolet B (UVB) radiation and particulate matter, which are especially potent external influencers, incites abnormal inflammatory reactions, ultimately disturbing epidermal homeostasis^7,8^. Notably, particulate matter exposure instigates TNF-α production via the aryl hydrocarbon receptor pathway, thereby impairing the skin barrier function and downregulating filaggrin in vitro and in vivo^11^.

Among environmental influences, UV radiation stands out as the most formidable factor driving skin-aging mechanisms, notably manifesting in processes like barrier impairment. Exposure of the skin to UVB radiation can lead to heightened generation of reactive oxygen species, thereby triggering inflammation through the activation of proinflammatory cytokines, including IL-1β, IL-6, IL-8, and TNF-α. UVB-irradiated keratinocytes modify the expression of epidermal differentiation specific markers, including keratin 10, loricrin, involucrin, and filaggrin^12,13^. Keratinocytes also alter the expression of proliferation markers in response to UVB irradiation^14,15^. Although substantial research endeavors have been directed toward exploring strategies for mitigating UV-induced cell damage, studies focused on addressing UVB-induced perturbations to epidermal homeostasis dysfunction remain scarce.

β-endorphin has been suggested as a key molecule for analgesia and in the reward and reinforcement associated with addiction^16,17^. β-endorphin can affect the peripheral system as well as the central nervous system by binding to the µ-opioid receptor^18^. β-Endorphin has the ability to inhibit excessive immune responses in the peripheral system^18,19^ and has been shown to specifically suppress the expression of IL-1β and TNF-α in synovial inflammatory tissues in a rat model of collagen-induced arthritis^19^.

Furthermore, the administration of β-endorphin to mice following skin grafting delayed rejection of the transplanted tissue, suggesting that β-endorphin exerts immunosuppressive effects^20^. The activation of µ-opioid receptors downregulates cytokine production and T-cell proliferation, thereby preventing excessive inflammatory responses in pathological tissue^21^. Additionally, Bigliardi et al. reported that β-endorphin and its receptor, µ-opioid receptor, may be involved in the process of wound healing^22^. Following UVB exposure, epidermal keratinocytes reportedly generate endogenous opioid β-endorphins through post-translational modification of the proopiomelanocortin (POMC) propeptide, which is produced via a p53-mediated induction of the *POMC* gene^23,24^. However, the role of β-endorphin released from UVB-irradiated skin has not yet been fully determined. Thus, in this study, we aimed to decipher the contribution of β-endorphin to skin health using normal human keratinocytes (NHKs) and a skin equivalent tissue model.

## Results

### Inhibitory effect of β-endorphin on inflammatory responses and activated NF-κB signaling in UVB-irradiated NHKs

**Figure 1:**
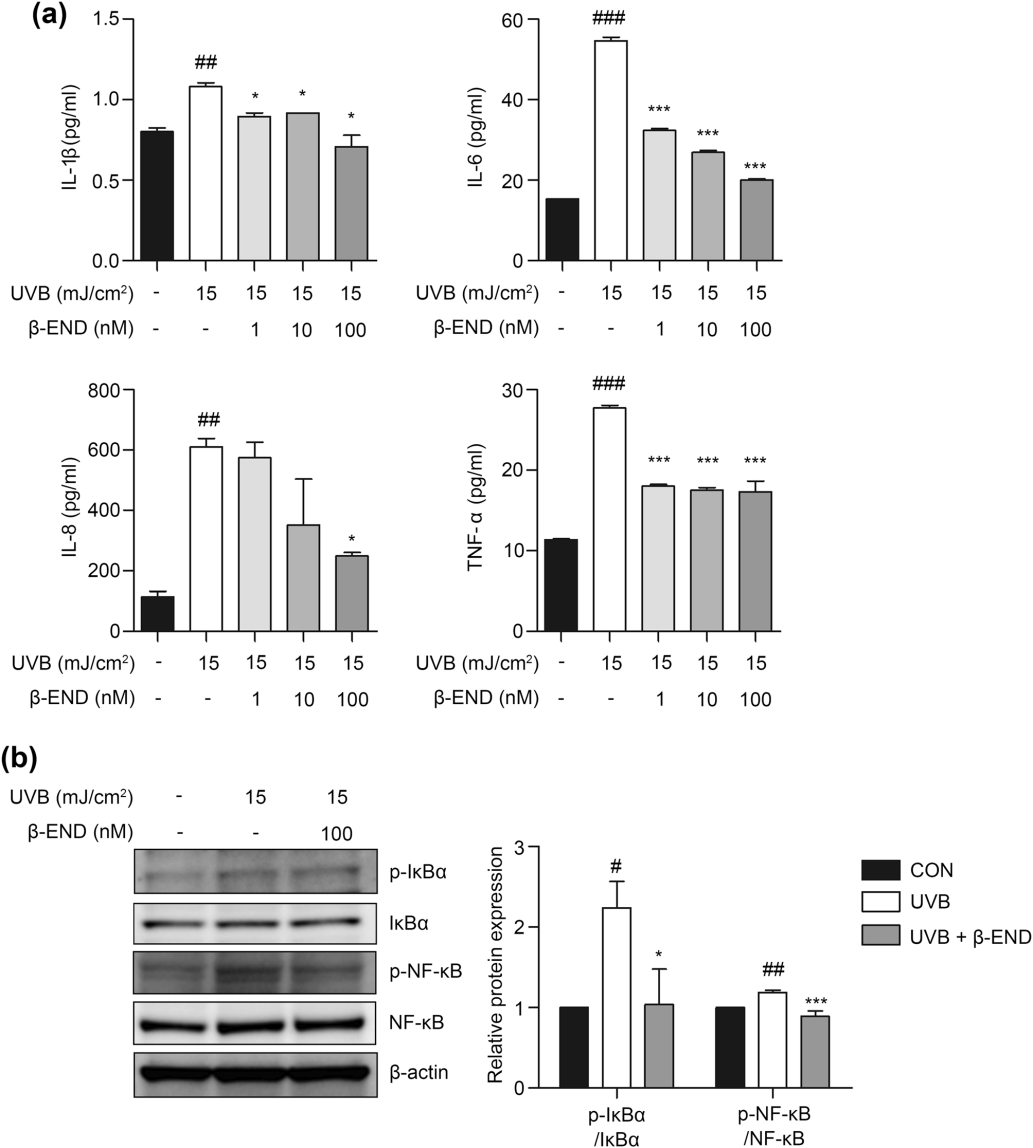
β-Endorphin inhibits the release of proinflammatory cytokines in NHKs triggered by UVB irradiation through the NF-κB signaling pathway. (**a**) Expression of secreted proinflammatory cytokines (interleukin (IL)-1β, IL-6, IL-8, and tumor necrosis factor (TNF)-α) was evaluated using the culture supernatant of NHKs after 15 mJ/cm^2^ UVB irradiation followed by 1, 10, and 100 nM β-endorphin treatment for 24 h. (**b**) Representative immunoblots showing the effects of β-endorphin on the NF-κB signaling pathway in the NHKs after 15 mJ/cm^2^ UVB irradiation followed by 24 h of 100 nM β-endorphin treatment. The original blots are presented in Supplementary Fig. S2. The phosphorylation levels of IκBα and NF-κB were analyzed using western blotting, and the quantification of the phosphorylation level of proteins is shown in the histogram. Data are presented as the mean ± SEM of six independent experiments. # *p* < 0.05, ## *p*<0.01, ### *p* < 0.001 compared to the non-irradiated group, and * *p* < 0.05, ** *p* < 0.01, *** *p* < 0.001 compared to the irradiated vehicle-treated group.

UVB irradiation mediates inflammatory responses that cause skin damage, including erythema, swelling, heat, pain, and skin barrier defects^12,25–27^. Given that skin β-endorphin levels are elevated in epidermal keratinocytes following UV exposure^24^, we hypothesized that β-endorphin protect against UVB-induced skin damage. To avoid an endogenous β-endorphin effect, we determined the UVB irradiation dose that did not induce an endogenous β-endorphin release. In our experimental system, low exposure to UVB (15 mJ/cm^2^ among 15, 20, and 25 mJ/cm^2^) irradiation in NHKs did not induce an endogenous β-endorphin release but did induce an inflammatory response (Fig. 1 and Supplementary Fig. S1). To determine the anti-inflammatory action of β-endorphin following UVB irradiation, NHKs were treated with β-endorphin following UVB irradiation for 24 h. Next, the secretion of proinflammatory cytokines was measured using an enzyme-linked immunosorbent assay (ELISA). The production of IL-1β, IL-6, IL-8, and TNF-α from keratinocytes increased following UVB irradiation and decreased in response to treatment with β-endorphin in a dose-dependent manner (Fig. 1a). For further investigation, we chose the 100-nM concentration of β-endorphin, which showed the maximum effect on suppression of cytokine release following UVB irradiation.

UVB irradiation activates NF-κB signaling pathways, contributing to proinflammatory cytokine secretion^28,29^. Because β-endorphin suppresses proinflammatory cytokine release, we examined whether β-endorphin affected the NF-κB signaling pathway activated by UVB in NHKs. Therefore, for the abovementioned reason, we chose the 100-nM concentration of β-endorphin for further investigation. After treating NHKs with β-endorphin following UVB irradiation for 24 h, the levels of proteins involved in the NF-κB signaling pathway were measured using western blotting analysis. Immunoblotting showed elevated levels of phospho(p)-IκBα and p-NF-κB in the UVB-only group. Meanwhile, β-endorphin treatment significantly decreased the expression of these proteins (Fig. 1b and Supplementary Fig. S2). These data indicate that the anti-inflammatory effect of β-endorphin in UVB-irradiated NHKs depends on NF-κB inactivation.

### Effect of β-endorphin on UVB-induced increase in proliferation marker levels and decrease in epidermal differentiation marker expression in NHKs

**Figure 2:**
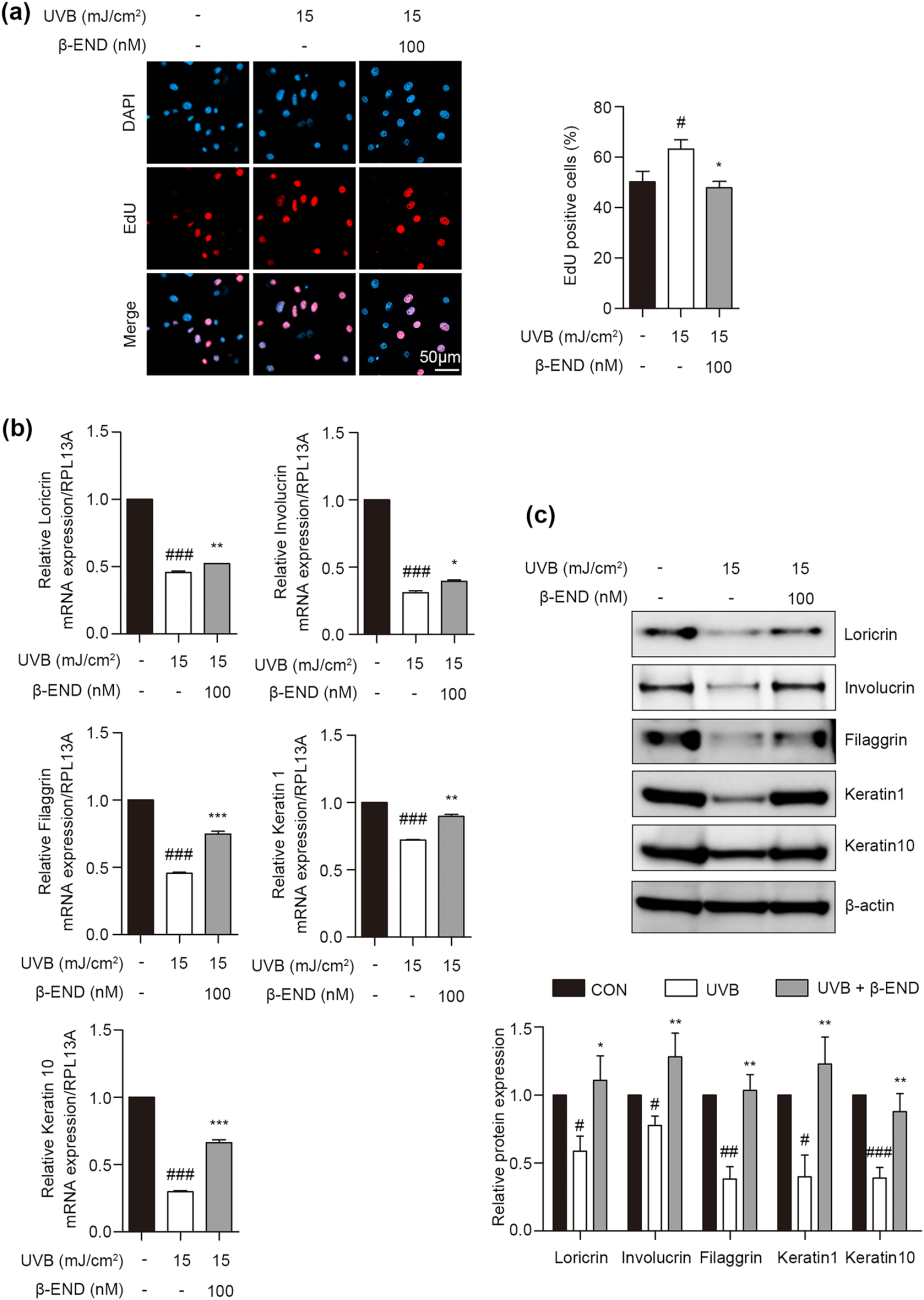
Treatment with β-endorphin salvaged UVB irradiation-induced increased proliferation and reduced the expression of epidermal differentiation markers in NHKs. (**a**) Representative images of 5-ethynyl-2ʹ-deoxyuridine (EdU)-positive cells in keratinocytes after UVB irradiation, followed by β-endorphin treatment for 24 h. DAPI: 4**′**,6-diamidino-2-phenylindole. The histogram shows the quantification of EdU as the percentage of cells with positive staining. (**b**) NHKs were exposed to 15 mJ/cm^2^ of UVB light, followed by 100 nM β-endorphin treatment for 48 h. RNA was isolated, and the mRNA expression of loricrin, involucrin, filaggrin, keratin 1, and keratin 10 was analyzed using RT-qPCR. Each mRNA level was normalized to that of the ribosomal gene ribosomal protein L13a (*RPL13A*). (**c**) Representative immunoblots showing differentiation markers expression levels. The original blots are presented in Supplementary Fig. S4. The protein expression levels of differentiation markers were determined via western blot analysis, and the quantification of these proteins is shown in the histogram. Data are presented as the mean ± SEM of six independent experiments. # *p* < 0.05, ##*p*<0.01, ### *p* < 0.001 compared to the non-irradiated group, and * *p* < 0.05, ** *p* < 0.01, *** *p* < 0.001 compared to the irradiated vehicle-treated group.

Several studies have proposed that UVB-induced inflammation disturbs the homeostasis of epidermal keratinocytes, leading to the exacerbation of skin diseases, such as those related to atopy^30–32^. We examined whether skin β-endorphin reversed the UVB-induced imbalance between the proliferation and differentiation in NHKs. In a proliferation assay, UVB-irradiated NHKs were treated with β-endorphin and 5-ethynyl-2′-deoxyuridine (EdU). Direct assessment of cellular proliferation using EdU labeling revealed increased numbers of EdU-positive nuclei, indicative of proliferation, in UVB-irradiated NHKs. Conversely, treatment with β-endorphin significantly reduced the number of EdU-positive nuclei (Fig. 2a and Supplementary Fig. S3). The suppression of UVB-induced excessive proliferation in keratinocytes by β-endorphin was corroborated by the results of the cell proliferation assay and decreased expression levels of PCNA (Supplementary Fig. S3). These data suggest that β-endorphin restore the abnormal proliferation caused by UVB irradiation in NHKs.

Furthermore, we investigated the protective role of β-endorphin in UVB-induced impairment of keratinocyte differentiation. UVB irradiation markedly reduced the expression of mRNAs and proteins involved in epidermal differentiation, such as loricrin, involucrin, filaggrin, keratin 1 and keratin 10 (Fig. 2b, c and Supplementary Fig. S4). Notably, treatment with β-endorphin significantly increased the expression levels of mRNA and proteins associated with epidermal differentiation, which were decreased following UVB irradiation (Fig. 2b, c and Supplementary Fig. S4). These findings indicate that β-endorphin alleviates the abnormal epidermal proliferation and differentiation of UVB-irradiated NHKs.

### Inhibitory effect of β-endorphin on Akt/mTOR signaling activated by UVB irradiation in NHKs

**Figure 3:**
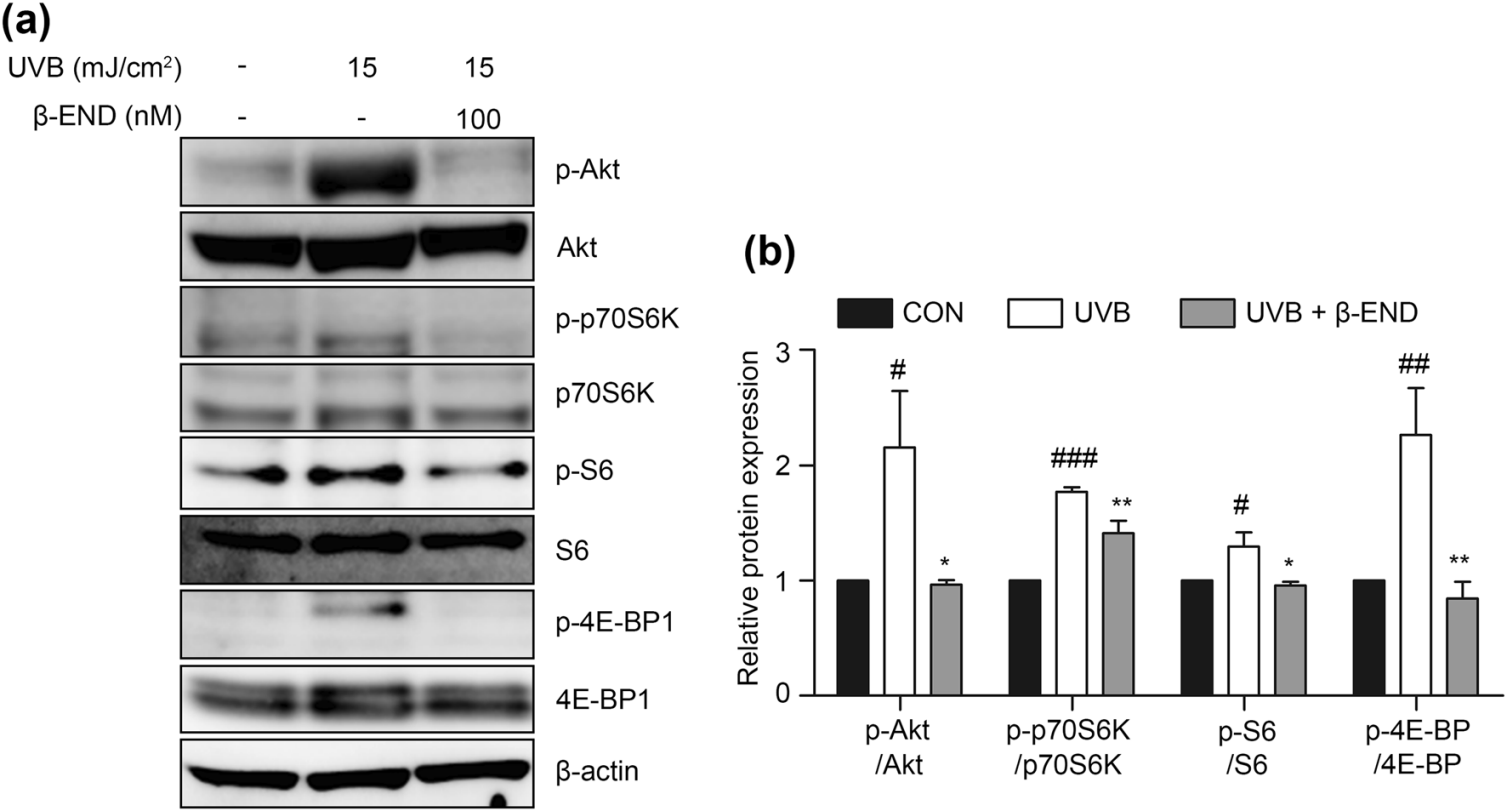
β-Endorphin inhibits the UVB irradiation-induced activation of the Akt/mTOR signaling pathway. (**a**) Representative immunoblots showing the effects of β-endorphin on the Akt/mTOR signaling pathway in NHKs after UVB irradiation followed by 100 nM β-endorphin treatment for 2 h. The original blots are presented in Supplementary Fig. S5. (**b**) The phosphorylation levels of the indicated proteins were analyzed via western blotting, and the quantification of the phosphorylation level of proteins is shown in the histogram. # *p* < 0.05, ##*p*<0.01, ### *p* < 0.001 compared to the non-irradiated group, and * *p* < 0.05, ** *p* < 0.01, *** *p* < 0.001 compared to the irradiated vehicle-treated group.

**Figure 4:**
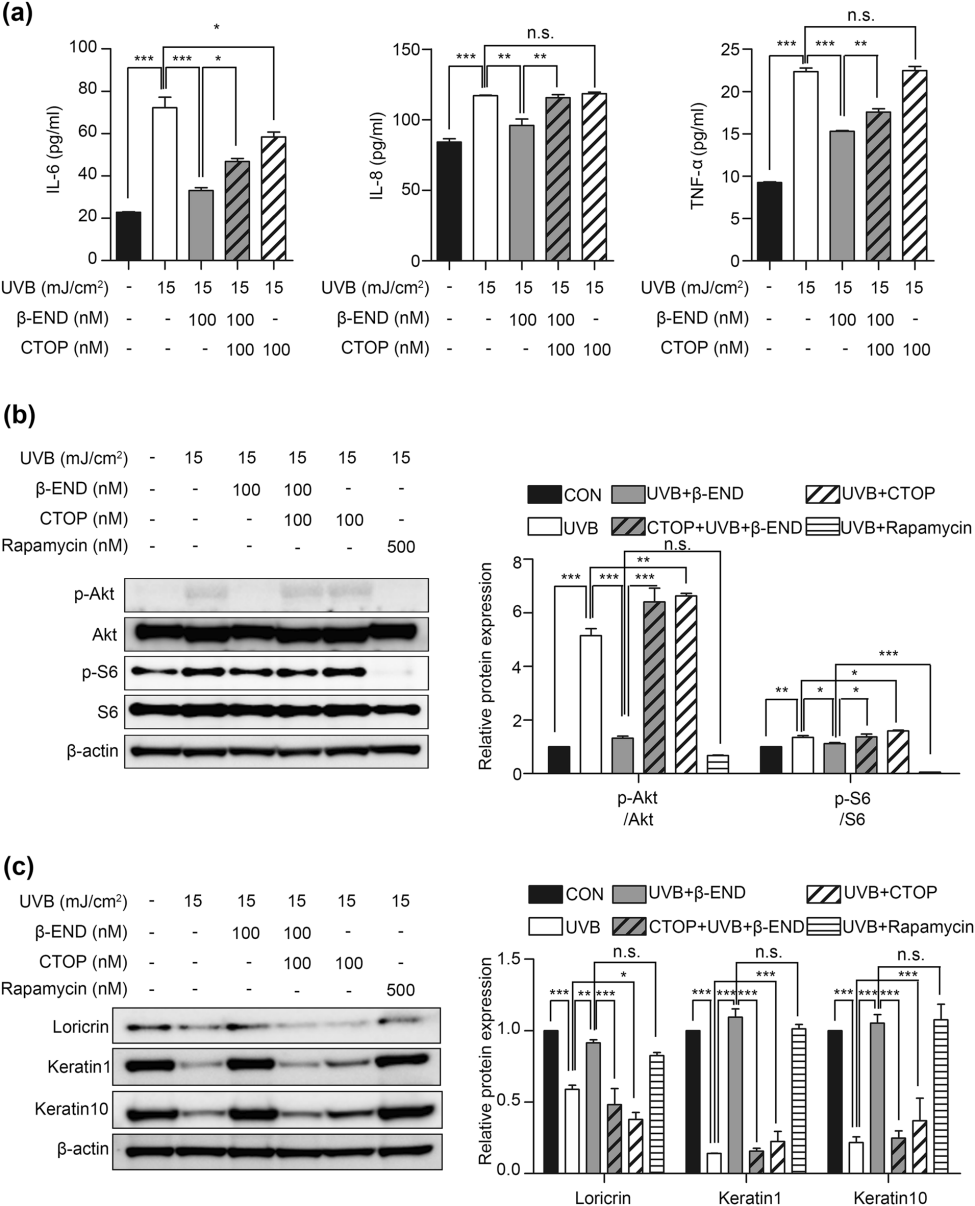
The effect of β-endorphin directly through the µ-opioid receptor, and the effect of inhibition of Akt/mTOR signaling on UVB-induced disruption of epidermal homeostasis. (**a**) NHKs were pre-incubated with 100 nM of CTOP for 30 min before 15 mJ/cm^2^ UVB irradiation and the addition of 100 nM β-endorphin. (**b**, **c**) Representative immunoblots and the quantification of the phosphorylation level of proteins in Akt/mTOR signaling pathway (**b**) and expression levels of differentiation markers (**c**) showing the reversal effect of β-endorphin is suppressed by 100 nM CTOP treatment and the reversal effect of 500 nM rapamycin treatment in NHKs for 30 min before 15 mJ/cm^2^ UVB irradiation. The original blots are presented in Supplementary Fig. S6. * *p* < 0.05, ** *p* < 0.01, *** *p* < 0.001 compared to the designated group. n.s. means not significant.

To further examine the molecular mechanism underlying the protective effect of β-endorphin against the disturbance in the balance between proliferation and differentiation caused by UVB irradiation, we conducted additional experiments. Proinflammatory cytokines induce Akt/mTOR signaling and interfere with keratinocyte differentiation by inducing proliferation^4,33^. To verify whether β-endorphin administration affects the Akt/mTOR signaling pathway, NHKs were exposed to UVB irradiation, followed by β-endorphin treatment for 2 h. Akt activation was shown by increased levels of p-Akt (Ser473), which indicate a maximal activation of Akt^34^. mTOR activation was demonstrated by increased levels of p-p70S6K (Thr389), p-S6 (Ser240/244), and p-4E-BP1 (Ser65). The immunoblotting results show that UVB irradiation induced significant activation of Akt/mTOR in NHKs (Fig. 3 and Supplementary Fig. S5). Notably, β-endorphin significantly reduced the phosphorylation of proteins in the Akt/mTOR signaling pathway. Furthermore, to ascertain whether β-endorphin directly counteracts UVB-induced inflammation and restores an epidermal homeostasis imbalance, we performed additional experiments using a β-endorphin receptor inhibitor. D-Phe-Cys-Tyr-D-Trp-Orn-Thr-Pen-Thr-NH2 (CTOP) is a selective antagonist of μ-opioid receptors and has widely been used to study the functions of β-endorphin^35^. We also used rapamycin, which is a known mTOR signaling inhibitor, for treatment to determine whether the inhibition of Akt/mTOR signaling could restore UVB-induced impairment of epidermal homeostasis. The reversal effects of β-endorphin were blocked by the μ-opioid receptor inhibitor CTOP (Fig. 4 and Supplementary Fig. S6). Moreover, inhibition of mTOR signaling using rapamycin restored epidermal homeostasis disruption by UVB (Fig. 4 and Supplementary Fig. S6). These findings suggest that β-endorphin markedly contributes to the protective effects against UVB-induced disruptions in epidermal homeostasis.

### Restoration of epidermal homeostasis by β-endorphin after UVB irradiation-induced barrier disruption in skin equivalents

**Figure 5:**
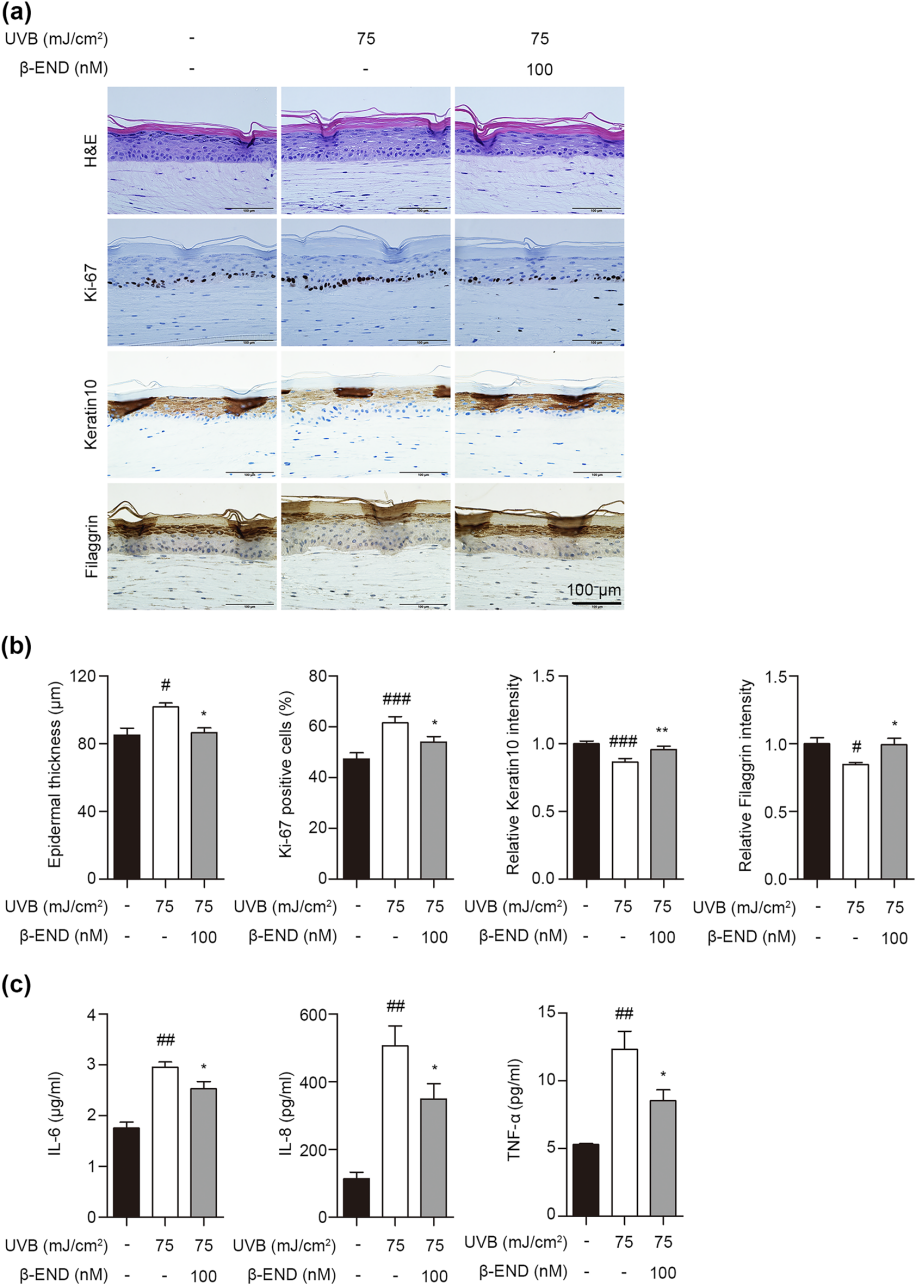
Protective effects of β-endorphin against UVB-induced epidermal damages. Skin equivalents were irradiated with UVB (75 mJ/cm^2^) followed by 100 nM β-endorphin for 48 h. (**a**) Representative images of histological sections of skin equivalents. Skin equivalent sections were stained with hematoxylin and eosin. The indicated proteins were detected using immunohistochemistry. Results are representative of three independent experiments. (**b**) Quantification of epidermal thickness, Ki-67 positive cells, and the expression levels of keratin 10, and filaggrin using ImageJ software. (**c**) The expression levels of proinflammatory cytokines IL-6, IL-8, and TNF-α were evaluated using the culture supernatant of skin equivalents after 75 mJ/cm^2^ UVB irradiation followed by 100 nM β-endorphin treatment for 48 h. Data in (**b**) and (**c**) are shown as the mean ± SEM of three independent fields obtained from three independent samples. # *p* < 0.05, ## *p* < 0.01, ### *p* < 0.001 compared to the non-irradiated group, and * *p* < 0.05, ** *p* < 0.01 compared to the irradiated vehicle-treated group.

Maintaining a balance between epidermal proliferation and differentiation is crucial for the proper functioning and homeostasis of the epidermis^36^. To address the regulatory effect of β-endorphin on UVB irradiation-induced disruption of epidermal homeostasis in human skin equivalents, skin equivalents were exposed to UVB and treated with β-endorphin for 48 h. UVB-irradiated skin equivalents showed hyperproliferative skin phenotypes: markedly elevated epidermal thickness and levels of Ki-67 proliferation markers (Fig. 5a, b and Supplementary Fig. S7). We also evaluated the intensity of immunostaining for epidermal differentiation markers (keratin 10 and filaggrin). UVB-irradiated skin equivalents showed decreased expression of epidermal differentiation markers. Notably, treatment with β-endorphin significantly reduced epidermal hyperproliferation and salvaged the expression of decreased keratinocyte differentiation markers in the UVB-irradiated skin equivalents (Fig. 5a, b and Supplementary Fig. S7).

We discovered that disruption of epidermal homeostasis was mediated by UVB irradiation-induced inflammation in NHKs. Next, we performed a cytokine array to evaluate whether treatment with β-endorphin suppresses cytokine production in human skin equivalents exposed to UVB irradiation. Treatment with β-endorphin on UVB-irradiated skin equivalents significantly reduced UVB irradiation-induced production of IL-6, IL-8, and TNF-α (Fig. 5c). Overall, these findings indicate that β-endorphin can reverse the UVB irradiation-induced imbalance between differentiation and proliferation by regulating the expression of inflammatory cytokines.

## Discussion

In this study, we demonstrated that β-endorphin protects against UVB-induced skin barrier dysfunction. We revealed that β-endorphin inhibits UVB irradiation-induced inflammation and restores the balance between the proliferation and differentiation that is disturbed by aberrant Akt/mTOR signaling activation.

Exacerbation of inflammatory responses, which leads to skin damage, occurs in skin diseases, including psoriasis, atopic dermatitis, and skin cancers, as well as in response to environmental stimuli, such as UV radiation and particulate matter^3,12,37^. UVB irradiation-induced inflammatory responses accelerate skin damage through the secretion of proinflammatory cytokines, including IL-1β, IL-6, IL-8, and TNF-α^38–40^. These proinflammatory cytokines are considered to be associated with the development of UVB-induced photodamage as well as epidermal dysfunction, such as hyperproliferation and abnormal differentiation. IL-1β stimulates the infiltration of immunocompetent and inflammatory cells and contributes to the pathogenesis of various skin diseases^41^. IL-6 effectively triggers keratinocyte proliferation, and its role has been studied in the wound healing process and in skin diseases, including psoriasis, correlated with epidermal hyperplasia^42,43^. IL-8, a chemokine that attracts neutrophils, is secreted from keratinocytes in response to external stimuli, including skin irritants. It triggers keratinocyte proliferation and has been detected in autoimmune diseases such as pemphigus herpetiformis^44^. TNF-α is a key agent in inflammatory disorders, including psoriasis and contact dermatitis, and mediates apoptosis, inflammation, and tumorigenesis^45^. In this study, under exposure to relatively low doses of UVB, NHKs and skin equivalents exhibited high levels of proinflammatory cytokines and disrupted the skin barrier. This finding suggests that exposure to low doses of UVB may result in an inflammatory microenvironment that induces proliferation and survival of malignant cells and promotes the development of skin tumors. β-endorphin has been suggested as an immunosuppressive modulator and cancer preventive in the periphery but has not been tested in the skin^46^. Here, we found that administration of β-endorphin resulted in the suppression of proinflammatory cytokine production in UVB-irradiated NHKs and skin equivalents.

The Akt/mTOR/NF-κB signaling pathway plays a crucial role in mediating the inflammatory response following UVB irradiation^47^. Toll-like receptors or IL-1β also promote inflammatory responses, including the release of these proinflammatory cytokines via activation of the Akt/mTOR/NF-κB signaling pathway^48,49^. Inflammation-induced Akt/mTOR/NF-κB activation has been observed in many inflammatory diseases, such as Crohn’s disease, celiac disease, atherosclerosis, and cardiovascular disease^50–52^. Additionally, hyperactivation of the Akt/mTOR pathway under inflammatory conditions occurs in the psoriatic epidermis; this dysregulation contributes to the pathogenesis of psoriasis^53,54^. Given that the mTOR pathway is critical in cellular proliferation and survival and that the imbalance between keratinocyte proliferation and differentiation leads to more severe skin pathologies, such as skin cancers, psoriasis, or atopic dermatitis, proper functioning of mTOR signaling is important for maintaining skin homeostasis and morphogenesis^52,53,55^. Blockage of mTOR through administration of rapamycin leads to a reduction in G1 to S cell cycle progression and hinders UVB irradiation-induced hyperproliferation of keratinocytes^33^. In addition, proper keratinocyte maturation occurs, as inhibition of mTOR facilitates differentiation progression^4^.

Therefore, the modulation of proinflammatory cytokine secretion and altered Akt/mTOR signaling is an attractive avenue to discover therapies for skin cancer and inflammatory skin diseases, including eczema, seborrheic dermatitis, and psoriasis. Our data show that treatment with β-endorphin inhibits the UVB-induced inflammatory response and normalizes epidermal homeostasis by inactivating Akt/mTOR signaling.

Endogenous β-endorphin is produced in response to skin UV irradiation through p53-mediated transcriptional induction of POMC and then through the post-translational cleavage of the POMC propeptide within the skin^24^. A previous study on endogenous β-endorphin release from UVB-irradiated keratinocytes focused only on its analgesic and psychological effects, not on UVB-irradiated skin itself^24^. Endogenous β-endorphin released from UV-exposed skin may suppress inflammation and protect the skin against UVB-induced damage through p53-dependent inactivation of the Akt/mTOR/NF-κB pathway^24,47,56^. In rats, systemic injection of β-endorphin has been shown to attenuate the increased immune response from collagen-induced arthritis by downregulating the NF-κB pathway^57^. Moreover, exogenous β-endorphin negatively regulates NF-κB by activating μ-opioid receptors^58,59^. In this study, we showed that exogenous β-endorphin inhibited UVB irradiation-induced secretion of proinflammatory cytokines, and this effect was abolished by treatment with the μ-opioid receptor antagonist CTOP. However, the mechanism by which β-endorphin normalizes epidermal homeostasis by regulating immune responses with μ-opioid receptors under UV irradiation has not been clearly elucidated in this study, and this should be explored in further studies.

A strength of this study is that it is the first study to indicate how β-endorphin affects skin health under UVB irradiation conditions, where endogenous β-endorphin is not secreted. Because endogenous β-endorphin was released from keratinocytes with UVB irradiation, it was difficult to determine the role of β-endorphin on UVB-irradiated skin; however, by carefully adjusting the irradiation condition of UVB, the role of β-endorphin was confirmed in conditions where β-endorphin were not secreted, but disruption of skin barrier occurred by UVB irradiation-induced inflammation. However, the limitation of this study is that the system we used did not include immune cells such as Langerhans and T cells. Although keratinocytes are known as a major source of the skin immune response, other immune cells are also involved in the skin inflammatory response^12^. Therefore, although the effectiveness of β-endorphin was verified using NHKs and skin equivalents herein, the effect of β-endorphin on human skin should be further validated.

In summary, our findings demonstrate that treating UVB-irradiated keratinocytes with β-endorphin leads to a significant reduction in the secretion of proinflammatory cytokines through the downregulation of NF-κB signaling. In addition, impaired epidermal homeostasis induced by UVB irradiation was normalized by β-endorphin via reversal of increased Akt/mTOR signaling. Furthermore, in our preliminary clinical data, we found that the expression level of β-endorphin in lesions of the epidermis in patients with inflammatory skin diseases (psoriasis, atopic dermatitis, and retinoid dermatitis) was lower than that in normal skin (Supplementary Fig. S8). However, further investigation is needed in the form of clinical studies with larger sample sizes to determine the levels of β-endorphin or the efficacy of β-endorphin treatment under excessive inflammation in the skin. Overall, these findings suggest that β-endorphin may have a potential application in the protection against skin damage caused by UVB-induced inflammation and could be a promising treatment for inflammatory skin disease.

## Methods

### Cell culture and treatment

Primary NHKs isolated from neonatal foreskin were purchased from Gibco (Carlsbad, CA, USA) and cultured in Epilife medium supplemented with 1% (v/v) human keratinocyte growth supplement (Cascade Biologics, Portland, OR, USA), 100 U/mL penicillin (Gibco), and 100 mg/mL streptomycin (Gibco) in a 5% CO_2_ incubator at 37 °C. Mycoplasma contamination testing was regularly performed using the MycoAlert® Mycoplasma detection kit (Lonza, Basel, Switzerland), following the manufacturer’s protocol. Keratinocytes were sub-cultured using Accutase® (Merck Millipore, Burlington, MA, USA) before reaching approximately 60–70% confluency, and cells passaged less than five times were used in the experiments. Keratinocytes were seeded in six-well plates, grown to approximately 90% confluence, and then exposed to UVB (15 mJ/cm^2^; Bio-Sun, Vilber Lourmat, Marne-la-Vallée, France). Immediately after UVB irradiation treatments, 1, 10, or 100 nM of β-endorphin (Sigma-Aldrich, St. Louis, MO, USA), CTOP (Tocris Bioscience, Bristol, UK), and rapamycin (Sigma-Aldrich), were added to the keratinocytes for various periods and at designated concentrations for each analysis.

### Quantitative real-time PCR (RT-qPCR)

Total RNA from keratinocytes was extracted with the RNeasy Mini kit (Qiagen, Chatsworth, CA, USA). After extraction, the concentration of total RNA was quantified and quality checked using a NanoDrop ND-1000 (NanoDrop Technologies, Wilmington, DE, USA). Thereafter, 1 µg of total RNA was used for complementary DNA (cDNA) synthesis using SuperScript VILO master mix (Life Technologies, Grand Island, NY, USA). Approximately 1 mg of cDNA samples and each TaqMan probe sets for keratin 1, keratin 10, filaggrin, loricrin, involucrin, and 60S ribosomal protein L13a (RPL13A) were purchased from Applied Biosystems (assay IDs: Hs00196158_m1, Hs00166289_m1, Hs00856927_g1, Hs01894962_s1, Hs00902520_m1, and Hs03043885_g1) and diluted in TaqMan universal master mix (Applied Biosystems, Foster City, CA, USA). Using a 7500 Fast Real-time PCR System (Thermo Fisher Scientific, Waltham, MA, USA), RT-qPCR was conducted. *RPL13A* was used to normalize target genes expression levels, and the fold difference was determined from threshold cycle values (2^−ΔΔCt^ method)^60^.

### Immunoblotting

Keratinocytes were lysed in RIPA buffer containing a protease and phosphatase inhibitor mixture (Sigma-Aldrich). Then, 30 µg of protein per sample was electrophoretically resolved on 4–12% NuPAGE gels (Invitrogen, Carlsbad, CA, USA) and transferred to a polyvinylidene difluoride membrane (Invitrogen). Proteins were probed with primary antibodies against involucrin (ab68), IκBa (ab32518), phospho-NF-κB (Ser536; ab86299), and NF-κB (ab32536) from Abcam (Cambridge, MA, USA); antibodies against keratin 1 (905204) and keratin 10 (905401) from BioLegend (San Diego, CA, USA); an antibody against filaggrin (PA5-116911) from Invitrogen; an antibody against loricrin (NBP1-33610) from Novus Biologicals (Littleton, CO, USA); an antibody against PCNA (sc-25280) from Santa Cruz Biotechnology (Santa Cruz, CA, USA); and the antibodies against phospho-IκBa (Ser32/36; 9246S), phospho-Akt (Ser473; 4060S), Akt (4691S), phospho-p70S6Kinase (Thr389; 9234S), p70S6Kinase (9202S), phospho-S6 (Ser240/244; 5364S), S6 (2217S), phosphor-4E-BP (Ser65; 9451S), 4E-BP (9644S), and β-actin (4967S) were obtained from Cell Signaling Technology (Beverly, MA, USA). Bands were probed with horseradish peroxidase-conjugated secondary antibodies (Invitrogen). The membranes were developed with ECL Plus (GE Healthcare, Chicago, IL, USA), and the immunoreactive bands were studied using an LAS-3000 imaging system (Fujifilm Life Science, Cambridge, MA, USA).

### ELISA for cytokine expression

Keratinocyte culture media were obtained and centrifuged at 400 × *g* for 5 min. The supernatant was used to measure cytokine levels. The secreted cytokine levels in the media were determined using the IL-1β, IL-6, IL-8, and TNF-α Duoset ELISA system (R&D Systems, Minneapolis, MN, USA), as described by the manufacturer.

### Immunofluorescence staining

Proliferation of keratinocytes was determined using the Click-iT EdU Imaging Kit (Invitrogen), following the manufacturer’s protocol. A total of 10 µM of EdU was added to the cells, which were incubated overnight. The cells were then fixed with 3.7% formaldehyde in phosphate-buffered saline (PBS), permeabilized with 0.5% TritonX-100 in PBS, and stained with fluorescent dye. Cell nuclei were stained with 4′,6-diamidine-2′-phenylindole dihydrochloride (DAPI) using ProLong™ Diamond Antifade Mountant with DAPI (Invitrogen). Confocal microscopy images of EdU-stained samples were obtained using a Zeiss LSM700 confocal microscope, and analysis of confocal microscopy images was performed using the Zen software (Carl Zeiss, Jena, Germany) for quantification.

### Skin equivalent model

Skin equivalents were prepared as previously described^61^. Normal human dermal fibroblasts (5 × 10^4^ cells per well; Lonza) were used to construct the dermal layer by mixing cell matrix type I (Nitta Gelatin Inc., Osaka, Japan) in reconstitution buffer (Nitta Gelatin Inc.). The mixture of fibroblasts was transferred to each insert of a six-well plate (Snapwell; Corning, NY, USA) and then incubated at 37 °C for 2 h for polymerization. The constructed dermal layer was cultured in human fibroblast expansion basal medium (Medium 106; Gibco) supplemented with 100 U/mL penicillin and 100 mg/mL streptomycin in a 5% CO_2_ incubator at 37 °C for 7 days. Keratinocytes (2 × 10^5^ cells per well) were seeded onto the constructed dermal layer to obtain skin equivalents. The reconstructed skin equivalents were immersed in Epilife medium (Gibco) for 1 day, then the medium was changed to the 3D culture medium CnT-3D-PR (CELLnTEC, Bern, Switzerland), and incubation continued for another day. The amount of media used was limited so that it could only reach the bottom layers of the skin equivalents, and they were subsequently exposed to air for 10 days to promote the differentiation of an epidermal layer. The skin equivalents were irradiated with UVB (75 mJ/cm^2^) and incubated with β-endorphin for 48 h. Afterward, the supernatants from skin equivalents were collected and the skin equivalents themselves were fixed with a 10% neutral buffered formalin solution (Sigma-Aldrich) and embedded into paraffin prior to analysis.

### Immunohistochemistry on skin equivalents

Paraffin-embedded samples were dewaxed in xylene and subsequently rehydrated through a series of descending ethanol–water mixtures (3 min per solution). Epitope retrieval was conducted for 10 min at 121 °C using Citrate Buffer pH 6.0 Antigen Retriever (64142; Electron Microscopy Sciences, Hatfield, PA, USA), and the slides were cooled before washing with 0.05% Tween 20 in PBS three times. The sections were then immersed in 1% H_2_O_2_ in water for 30 min to inactivate endogenous peroxidase and subsequently rinsed with water and 0.05% Tween 20 in PBS for 5 min. The blocking process was performed by incubating the slides with 10% goat serum (Dako, Carpinteria, CA, USA) in PBS for 20 min before exposure to the following primary antibodies overnight at 4 °C: anti-keratin 10 (905401; BioLegend), anti-filaggrin (ab81468; Abcam), and anti-Ki-67 (ab15580; Abcam). The samples were completely washed by immersing in water for 10 min and 0.05% Tween 20 in PBS for 5 min, followed by 30 min of incubation with horseradish peroxidase-conjugated anti-primary antibody (EnVision + Single Reagent, K4001 and K4003; Dako, Carpinteria, CA, USA). The samples were then washed in water and PBS before incubation with the Liquid DAB + Immunohistochemistry Visualization System (K3468; Dako) for 3–5 min. The reaction was terminated by submerging in water; counterstaining was performed for 3–5 min using Mayer’s hematoxylin (Dako). The sections were washed with water for 1 min and the signals were developed in Scott’s tap water substitute for 2 min. Subsequently, the sections were dehydrated in graded ethanol, cleared in xylene, and mounted using Cytoseal 60 Mountant (Thermo Fisher Scientific).

### Statistical analysis

All experiments were repeated at least three to six times. Data were evaluated using one-way analysis of variance followed by the Newman–Keuls multiple comparisons test to analyze statistical significance after confirming normality of data using the Shapiro–Wilk test. A *p* value less than 0.05 was considered statistically significant. All data are presented as the mean ± standard error.

### Supplementary Information

Supplementary Information.

## Data Availability

The datasets supporting the current study will be shared by the corresponding authors upon a reasonable request from any qualified investigator.
