# Supplementary material for: β-endorphin suppresses ultraviolet B irradiation-induced epidermal barrier damage by regulating inflammation-dependent mTORC1 signaling
Source: Sci Rep. 2023 Dec 15;13:22357. doi: 10.1038/s41598-023-49886-5 (PMC10724221; doi:10.1038/s41598-023-49886-5)
Supplement: Supplementary file 1 — Supplementary Information. [file 41598_2023_49886_MOESM1_ESM.pdf]

Supplementary Information

β-Endorphin Suppresses Ultraviolet B Irradiation-Induced Epidermal Barrier Damage by Regulating Inflammation-Dependent mTORC1 Signaling

Hyung-Su Kim, Hyoung-June Kim, Yong-Deog Hong, Eui Dong Son, and Si-Young Cho

Supplementary Figures S1–S8.

Supplementary Methods

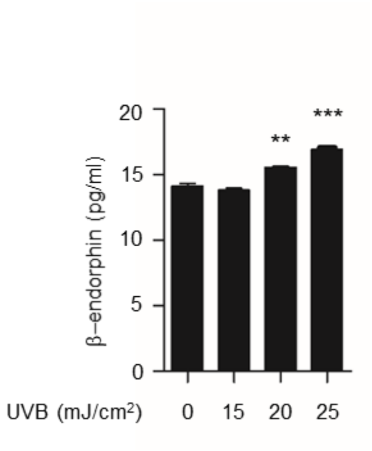

Supplementary Figure S1. β-Endorphin is not secreted from normal human keratinocytes (NHKs) by UVB irradiation of 15 mJ/cm^2. ** p < 0.01, *** p < 0.001.

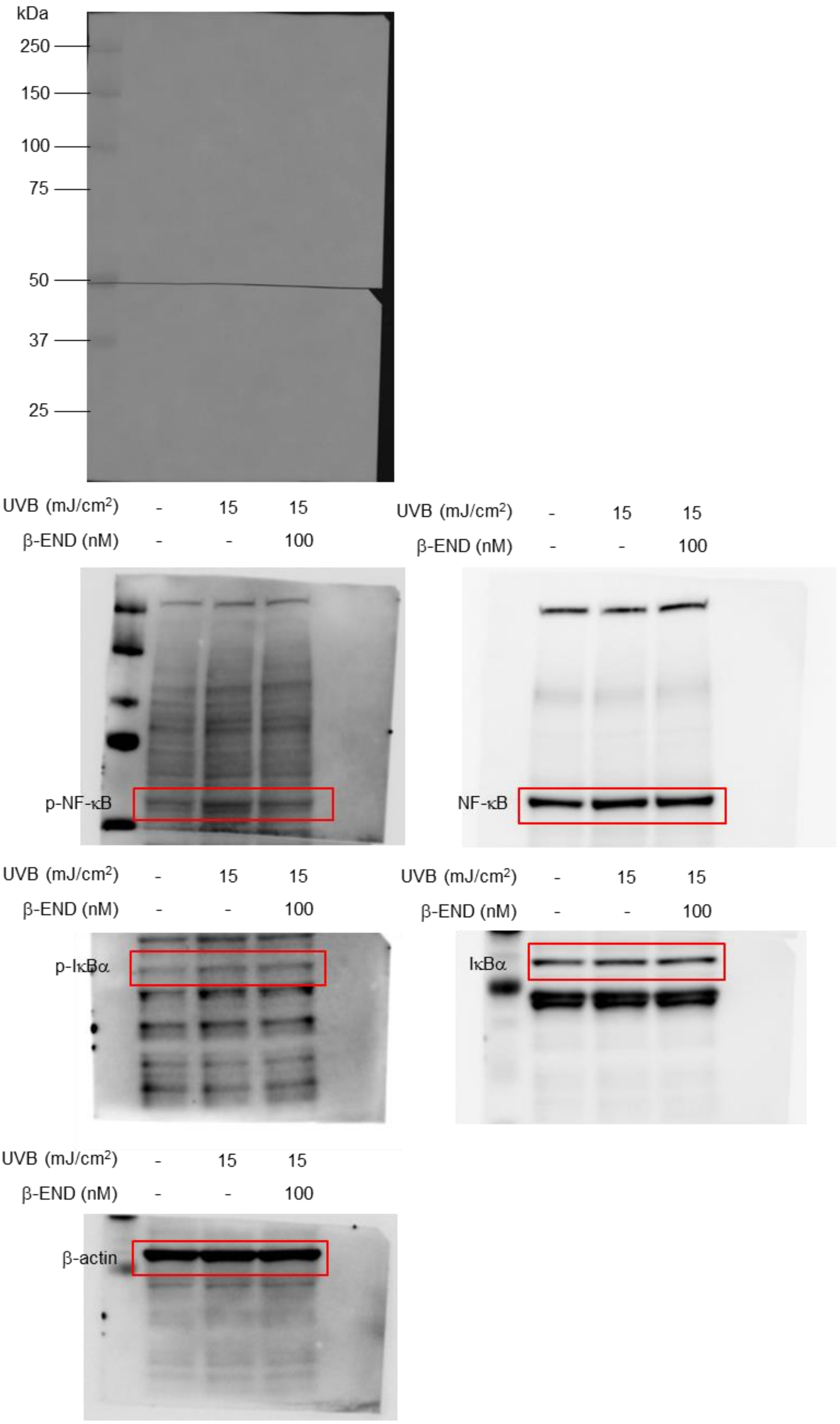

Supplementary Figure S2. The full-blotting data of β-endorphin treatment for NF-κB signaling proteins in UVB-irradiated NHKs.

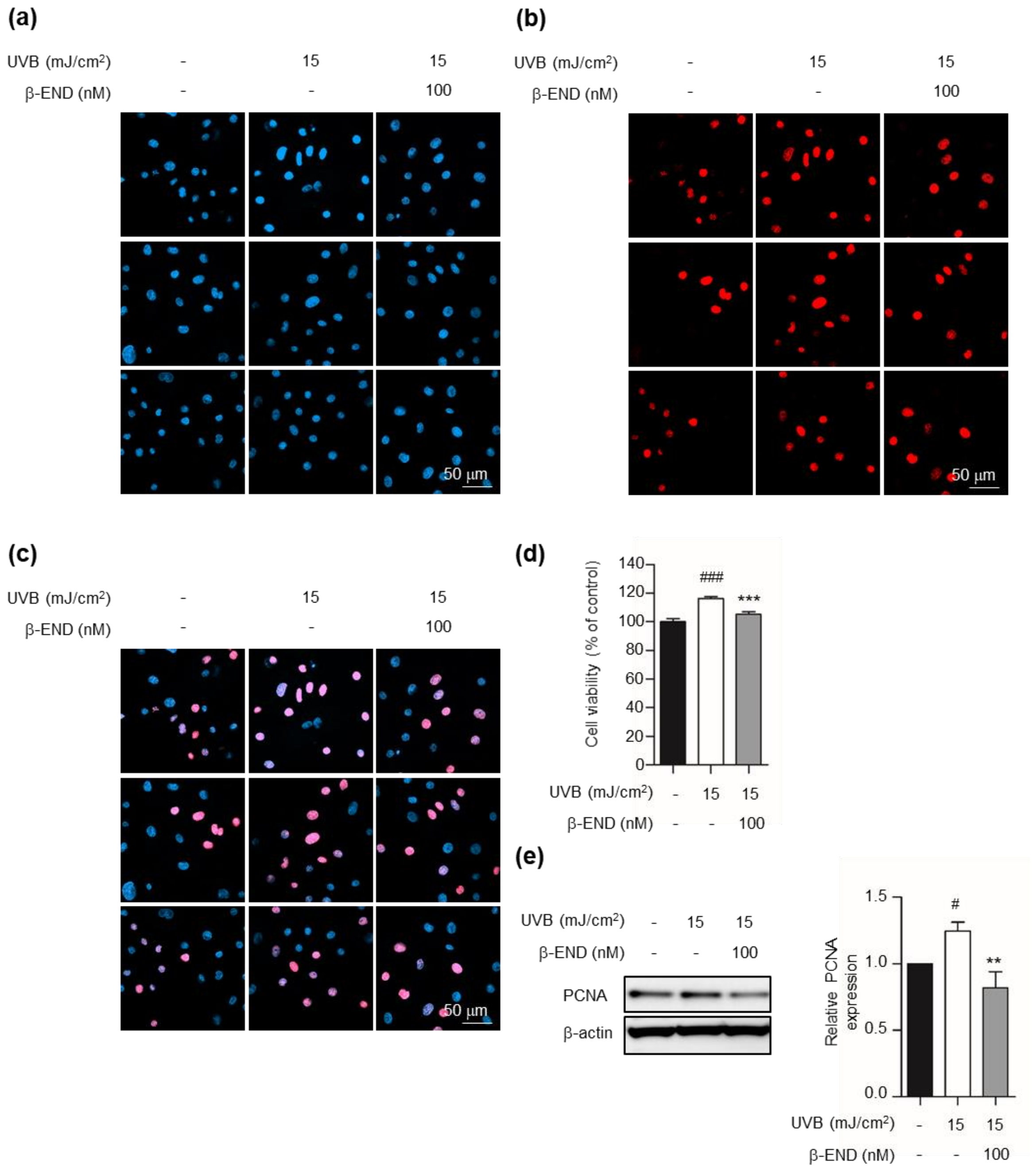

Supplementary Figure S3. Treatment with β-endorphin salvages UVB-induced increased proliferation of NHKs. The representative data are displayed in Figure 2a. (a) DAPI. (b) EdU. (c) Merged images of DAPI and EdU. (d) Cell viability of NHKs after UVB irradiation followed by β-endorphin treatment. (e) Representative immunoblots showing the effect of β-endorphin on the inhibition of UVB-induced increments of PCNA expression. # $p < 0.05$, ### $p < 0.001$ compared to the non-irradiated group, and ** $p < 0.01$, *** $p < 0.001$ compared to

the irradiated vehicle-treated group.

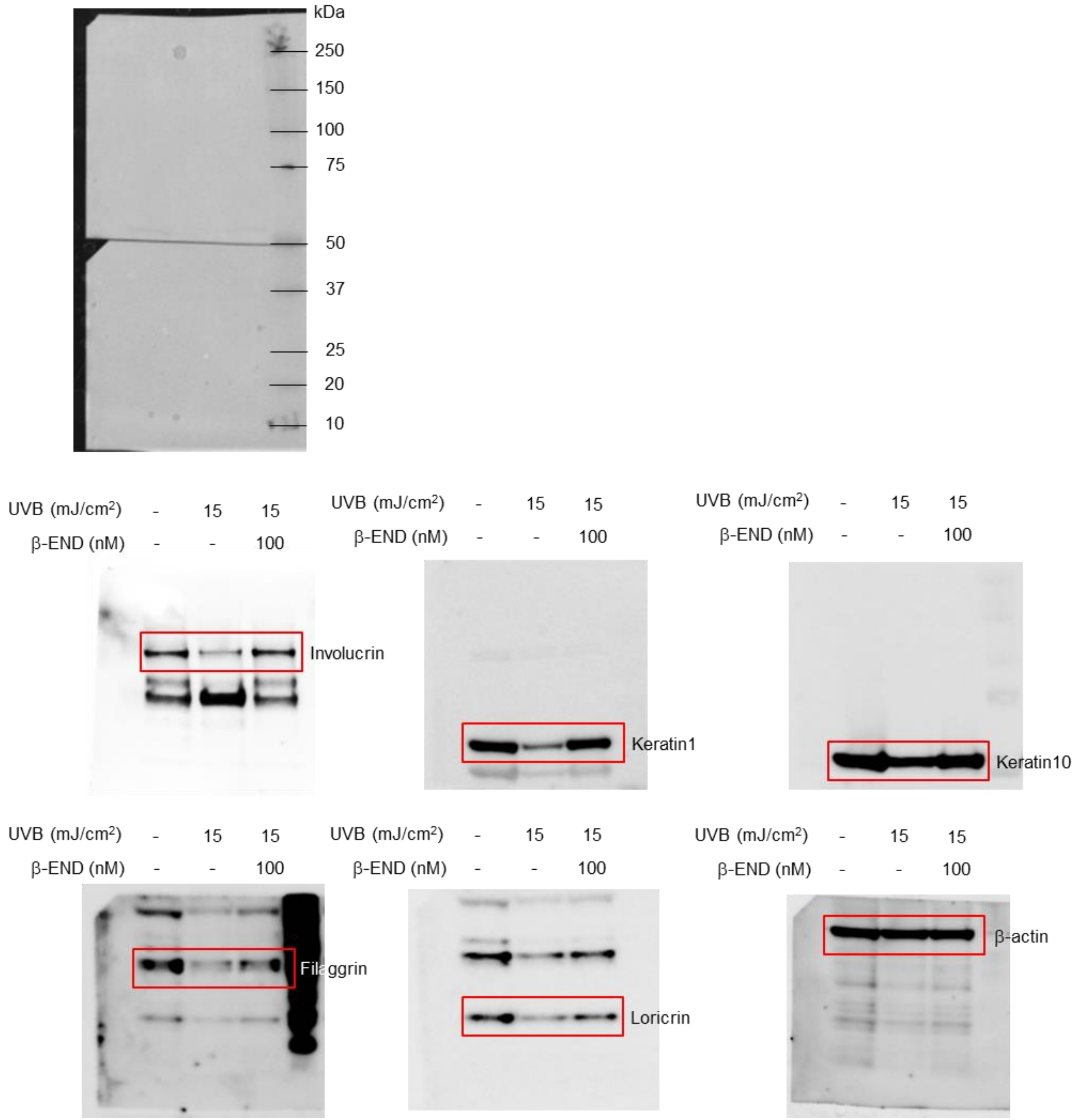

Supplementary Figure S4. The full-blotting data of β-endorphin treatment for differentiation markers in UVB-irradiated NHKs.

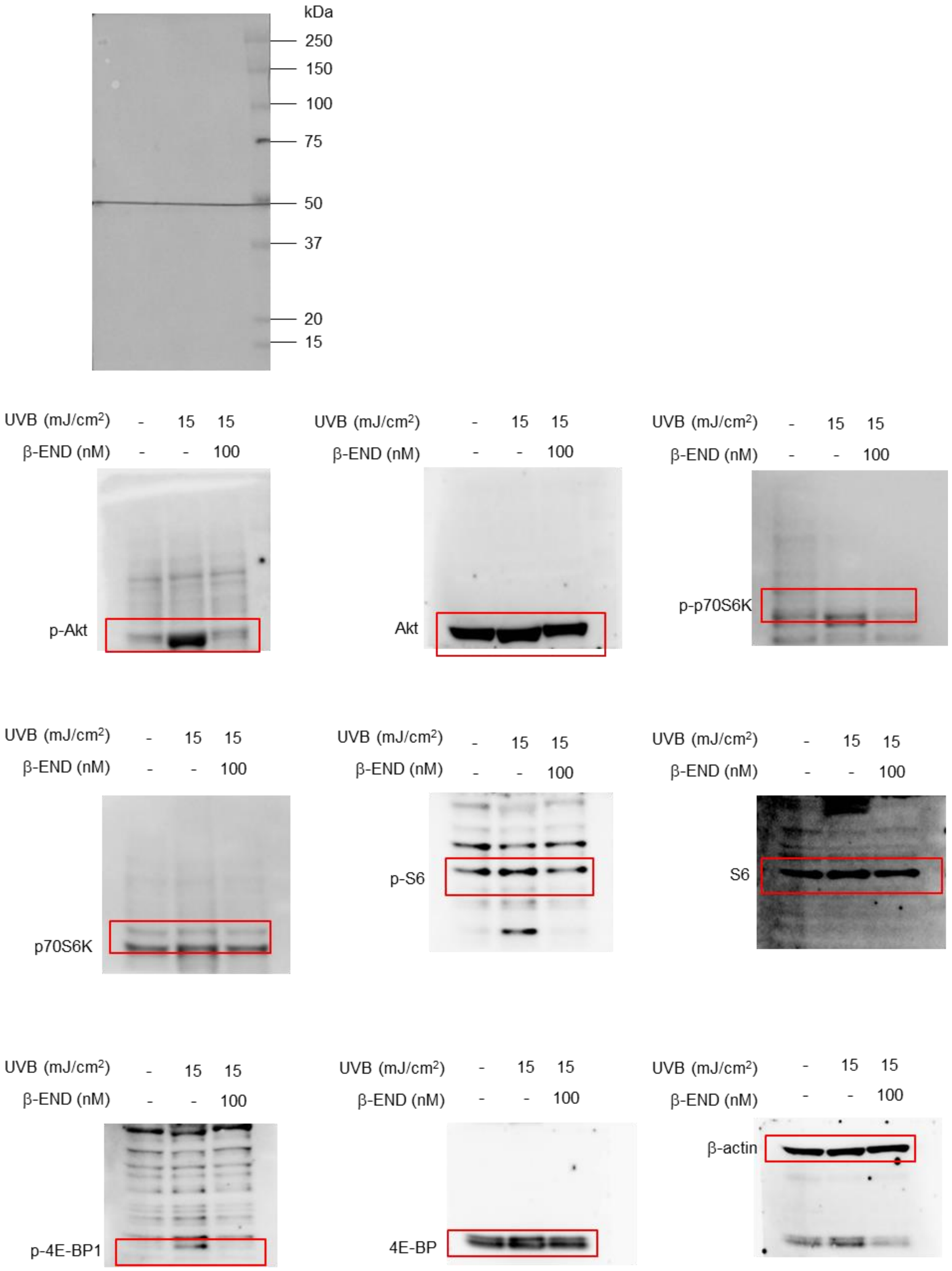

Supplementary Figure S5. The full-blotting data of β-endorphin treatment Akt/mTOR signaling proteins in UVB-irradiated NHKs.

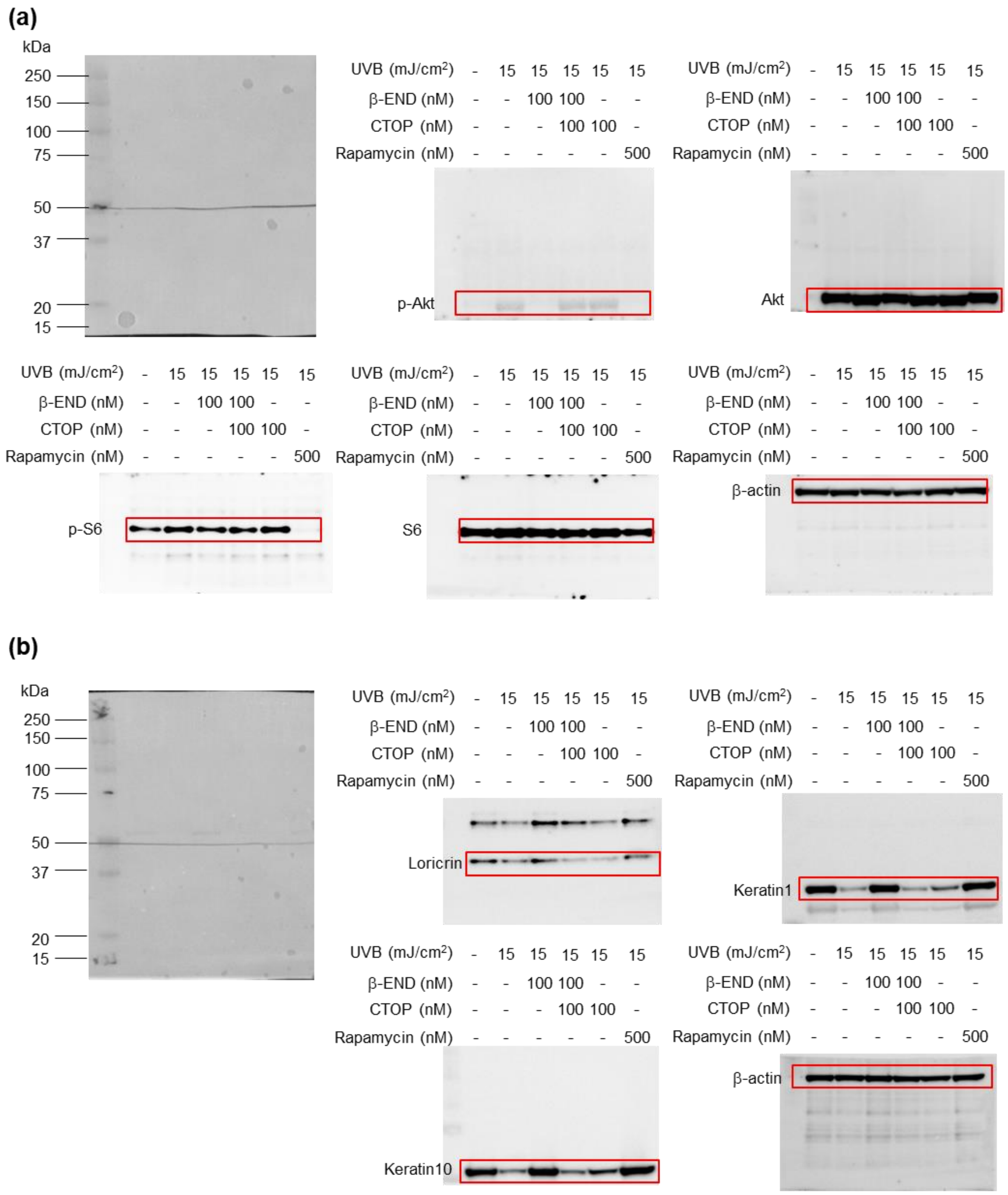

Supplementary Figure S6. The full-blotting data of the effect of β-endorphin on Akt/mTOR signaling protein levels (a) and differentiation markers (b) was abolished by CTOP treatment, and the effect of rapamycin in UVB-irradiated NHKs.

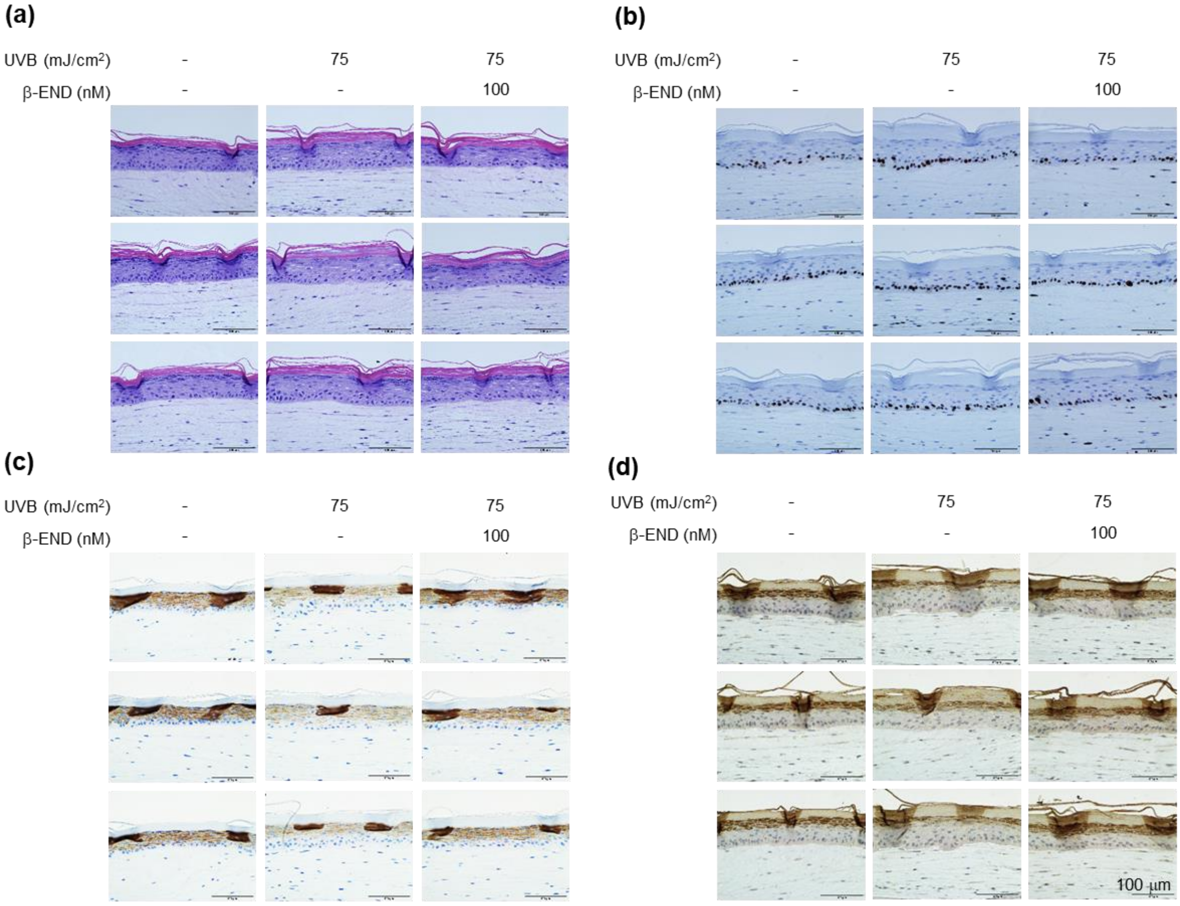

Supplementary Figure S7. Effect of β-endorphin on UVB irradiation-induced proliferation and expression of differentiation-related genes in skin equivalents. The representative data are displayed in Figure 5A. (a) Hematoxylin and eosin. (b) Ki-67. (c) Keratin 10. (d) Filaggrin. Scale bars: 100 μm.

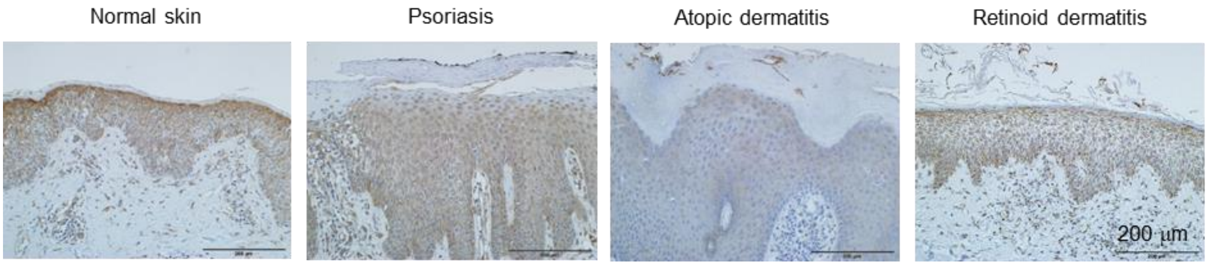

Supplementary Figure S8. β-endorphin expression decreases in lesional skin of psoriasis, atopic dermatitis, and retinoid dermatitis compared to that in normal skin. In normal skin, β-endorphin was expressed in the epidermis and mostly located in the subcorneal layers of the epidermis. In lesional skin of psoriasis, atopic dermatitis, and retinoid dermatitis, this pattern of β-endorphin expression was disrupted. Scare bar: 200 μm

Supplementary Methods

Measurement of β-endorphin release

Normal human keratinocytes (NHKs) were grown in a 35-mm dish and exposed to UVB (15, 20, and 25 mJ/cm^2; Bio-Sun, Vilber Lourmat, Marne-la-Vallée, France). Each plate contained 1 ml of media for the release assay. Six hours after irradiation, β-endorphin content in the supernatant was measured using a commercially available enzyme immunoassay following the manufacturer's instruction (Pheonix pharmaceuticals, Belmont, CA, USA).

Cell viability analysis

NHKs were seeded and grown in 24-well plate and exposed to 15 mJ/cm^2 UVB followed by β-endorphin treatment. Six hours after irradiation, MTS colorimetric cell proliferation assay (Promega, Madison, WI, USA) was performed to quantify cell proliferation following the supplier's instructions.

Immunohistochemical staining of preliminary clinical sample

A patient with atopic dermatitis, two with psoriasis, one with retinoid dermatitis, and two non-patient controls were included in the study. All treatments and sample acquisitions were approved by the Institutional Review Board of Korea University Hospital (IRB: 2020AS0178). The study was conducted according to the Declaration of Helsinki. Informed written consent was obtained for all procedures. Patients with psoriasis, atopic dermatitis, and retinoid dermatitis did not receive topical therapy or systemic therapy, including biologics, for at least 4 weeks prior to the study. Punch biopsies with a 4-mm diameter were obtained from the lesional skin of atopic dermatitis, retinoid dermatitis, psoriasis skin, or normal lower back skin of healthy controls. The punch biopsy specimens were fixed with 10% neutral

buffered formalin, and then processed into paraffin blocks. The antibody used to stain the β-endorphin was a commercially available, affinity purified, polyclonal rabbit anti-β-endorphin antibody (Biorbyt, Cambridge, UK). The sections were incubated with primary antibody for 16 h at 4 °C.
